# Environmental Impact on Differential Composition of Gut Microbiota in Indoor Chickens in Commercial Production and Outdoor, Backyard Chickens

**DOI:** 10.3390/microorganisms8050767

**Published:** 2020-05-20

**Authors:** Zuzana Seidlerova, Tereza Kubasova, Marcela Faldynova, Magdalena Crhanova, Daniela Karasova, Vladimir Babak, Ivan Rychlik

**Affiliations:** Veterinary Research Institute, 62100 Brno, Czech Republic; seidlerova@vri.cz (Z.S.); kubasova@vri.cz (T.K.); faldynova@vri.cz (M.F.); crhanova@vri.cz (M.C.); karasova@vri.cz (D.K.); babak@vri.cz (V.B.)

**Keywords:** chicken microbiota, microbiome, caecum, environment, backyard chicken

## Abstract

In this study, we compared the caecal microbiota composition of egg-laying hens from commercial production that are kept indoors throughout their whole life with microbiota of hens kept outdoors. The microbiota of outdoor hens consisted of lower numbers of bacterial species than the microbiota of indoor hens. At the phylum level, microbiota of outdoor hens was enriched for Bacteroidetes (62.41 ± 4.47% of total microbiota in outdoor hens and 52.01 ± 6.27% in indoor hens) and Proteobacteria (9.33 ± 4.99% in outdoor and 5.47 ± 2.24% in indoor hens). On the other hand, Firmicutes were more abundant in the microbiota of indoor hens (33.28 ± 5.11% in indoor and 20.66 ± 4.41% in outdoor hens). Horizontally transferrable antibiotic resistance genes *tetO*, *tet(32)*, *tet(44)*, and *tetW* were also less abundant in the microbiota of outdoor hens than indoor hens. A comparison of the microbiota composition at the genus and species levels pointed toward isolates specifically adapted to the two extreme environments. However, genera and species recorded as being similarly abundant in the microbiota of indoor and outdoor hens are equally as noteworthy because these represent microbiota members that are highly adapted to chickens, irrespective of their genetics, feed composition, and living environment.

## 1. Introduction

Chickens in commercial production are hatched without any contact with adult birds, and colonization of their intestinal tract is dependent on environmental sources only. The development of their microbiota is therefore slower than in chicks that have any form of contact with adult hen microbiota [1,2]. The speed of microbiota development is quite important since the level of microbiota development correlates with the chicken’s natural resistance to infection with pathogens like Salmonella or Clostridium perfringens [3,4,5]. Both the speed and quality of microbiota establishment is therefore key for the production of healthy chickens and, consequently, for improved productivity, reduced need for antibiotic therapy, and increased animal welfare [6,7].

Attempts with administration of microbiota from healthy adults to newly hatched chicks in intensive production systems date back to the 1970s [2]. The concept, termed competitive exclusion due to the exclusion of enteric pathogens from gut colonization, is known in poultry production. Products containing complex gut microbiota obtained by anaerobic culture of fecal microbiota from adult hens, e.g., Aviguard or Broilact, are commercially available. However, hens used as donors of fecal microbiota for the fermentation of competitive exclusion products are hatched in hatcheries, without any contact with microbiota from healthy adult hens. Moreover, to minimize the risk of uncontrolled infection of the donor hens, these hens are kept under highly contained conditions. However, this style of raising chickens is quite different from the conditions to which chickens have adapted during millions of years of evolution. While a contained environment is necessary for the production of safe products, this measure may exclude some beneficial microbes from entering the intestinal tract of these donor hens. This is why we have become interested in the differences in microbiota of indoor hens in intensive commercial production and outdoor chickens kept in backyards of private owners for their own egg production.

**Figure 1:**
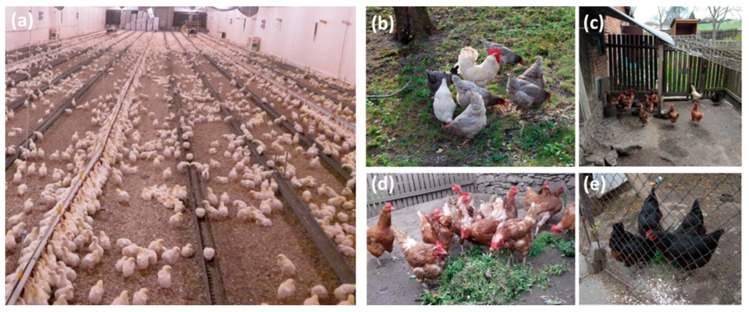
Living environment of indoor and outdoor chickens. Raising of egg layers in commercial production in an indoor environment (panel (**a**)) and egg laying hens in an outdoor, backyard environment (panels (**b**–**e**)).

In this study, we therefore compared the caecal microbiota composition in chickens from commercial production, kept indoors throughout their whole life and with microbiota of chickens kept outdoors throughout their whole life. Caecal microbiota was selected due to its high complexity and also importance for natural resistance of chickens to the colonization of pathogens like Salmonella. Private owners in village areas of the Czech Republic keep small flocks of 5–10 hens for their own egg consumption, i.e., not for global market distribution (Figure 1). This comparison allowed us to address the importance of the external environment in shaping chicken gut microbiota. Due to access to a wide variety of feed, we expected that the microbiota of outdoor chickens would be more complex than the microbiota of commercial indoor chickens. However, what if the feed of commercial indoor chickens enriched for vitamins, trace elements, specific feed additives etc. may allow for a broader microbiota to colonize their intestinal tract? Could the outdoor chicken’s specific microbiota members be used as probiotics for indoor chickens, or do they represent microbiota members too adapted to outdoor chickens? Or are the microbiota members present in both indoor and outdoor chickens those of the highest probiotic potential since these are always present, irrespective of chicken genetic line, different feed, and habitat occupied? Though not all these questions could be ultimately answered in a single study, a comparison of microbiota of outdoor and indoor chickens is the first necessary step towards a better understanding of chicken core microbiota and microbiota members specifically selected under two extreme environmental ecosystems.

## 2. Materials and Methods

### 2.1. Experimental Animals

Twenty 30–50-week-old hens were obtained from three different egg producing farms using enriched battery cages for egg production. At any time of rearing or egg production, these hens had no access to the outdoor environment and were kept in a strictly controlled indoor environment. An additional 17 hens were obtained from six different backyard poultry flocks with the agreement of the owners. These hens were 2–4 years old and were reared year-round in an outdoor environment with access to a simple henhouse. Both indoor and outdoor hens were brought to the post-mortem room, humanely sacrificed, and their caecal contents were collected and frozen at −70 °C. The time from euthanization to sample freezing never exceeded 30 min and time between sample freezing and DNA purification never exceeded three months.

The handling of animals in the study was performed in accordance with current Czech legislation (Animal Protection and Welfare Act No. 246/1992 Coll. of the Government of the Czech Republic). The specific experiments were approved by the Ethics Committee of the Veterinary Research Institute followed by the Committee for Animal Welfare of the Ministry of Agriculture of the Czech Republic (permit number MZe1922 approved on January 15, 2018).

### 2.2. Sequencing of V3/V4 Region of 16S rRNA Genes

Caecal content samples were homogenized in a MagNALyzer (Roche, Prague, Czech Republic). Following homogenization, the DNA was extracted using a QIAamp DNA Stool Mini Kit according to the manufacturer’s instructions (Qiagen, Hilden, Germany). The DNA concentration was determined spectrophotometrically, and DNA samples diluted to 5 ng/mL were used as a template in PCR with forward primer 5′-*TCGTCGGCAGCGTCAGATGTGTATAAGAGACAG*-MID-GT-CCTACGGGNGGCWGCAG-3′ and reverse primer 5′-*GTCTCGTGGGCTCGGAGATGTGTATAAGAGACAG*-MID-GT GACTACHVGGGTATCTAATCC-3′. The sequences in italics served for index ligation whereas the underlined sequences allowed for amplification over the V3/V4 region of 16S rRNA genes. MIDs represent different sequences of 5, 6, 7, or 9 base pairs in length, which were used to identify individual samples after the whole sequencing run. PCR (Polymerase Chain Reaction) amplification was performed using a KAPA HiFi Hot Start Ready Mix kit (Roche, Basel, Switzerland), and the resulting PCR products were purified using AMPure beads (Beckman Coulter, Prague, Czech Republic). In the next step, the PCR product concentration was determined spectrophotometrically, the DNA was diluted to 100 ng/μL, and groups of 14 PCR products with different MID sequences were indexed with the same index from Nextera XT Index Kit following the manufacturer’s instructions (Illumina, San Diego, CA, USA). The next set of 14 PCR products with different MID sequences were indexed with the next index from the Nextera XT Index kit, thus allowing an increase in the number of samples analyzed in a single sequencing run. Prior to sequencing, the concentration of differently indexed samples was determined using a KAPA Library Quantification Complete kit (Roche, Basel, Switzerland). All indexed samples were diluted to 4 ng/μL, and 20 pM phiX DNA was added to a final concentration of 5% (v/v). Sequencing was performed using MiSeq Reagent Kit v3 (600 cycle) and MiSeq apparatus according to the manufacturer’s instructions (Illumina, San Diego, CA, USA). Quality trimming of the raw reads was performed using TrimmomaticPE v0.32 with a sliding window of 4 bp and a quality read score equal or higher than 20 [8]. Minimal read length must have been at least 150 bp. The fastq files generated after quality trimming were uploaded into QIIME software [9]. Forward and reverse sequences were joined, and in the next step, chimeric sequences were predicted and excluded by the slayer algorithm. The resulting sequences were then classified by RDP Seqmatch with an operational taxonomic unit (OTU) discrimination level set to 97%. Principal coordinate analysis (PCoA) implemented in QIIME was used for data visualization.

### 2.3. Real-Time PCR Quantification of Antibiotic Resistance Genes and Genes Specific for Representatives of Selected Genera

Real-time PCR was used for the quantification of *linA*, *tetA(P)*, *tetQ*, *tetO*, *tet(32)*, *tet(44)*, tetW, *bla_TEM_*, *aadA*, *strA*, and *sul2* genes, as well as 10 selected microbiota members, including *Anaerotruncus colihominis*, *Pseudoflavonifractor capillosus*, *Butyricicoccus pullicaecorum*, *Flavonifractor plautii*, *Olsenella uli*, *Bacteroides mediterraneensis*, *Bacteroides coprophilus*, *Mucispirillum schaedlerii*, and *Marseilla massiliensis*. PCR was performed in 3μL volumes in 384-well microplates using QuantiTect SYBR Green PCR Master mix (Qiagen). Dispensing of the PCR master mix, primers, water and DNA was performed using a Nanodrop pipetting station (Innovadyne, Santa Rosa, CA, United States). PCR and signal detection were performed with a LightCycler II (Roche) with an initial denaturation at 95 °C for 15 min followed by 40 cycles of denaturation at 95 °C for 20 s, primer annealing at 60 °C for 30 s, and extension at 72 °C for 30 s. Each sample was subjected to real-time PCR in triplicate and the mean values of the triplicates were used for subsequent analysis. Amplification of the 16S rRNA gene using Eubacteria specific primers was used as a reference to determine the total amount of bacterial DNA in each sample (for primer sequences, see Table S1). Ct values of genes of interest were subtracted from the Ct value of eubacterial 16S rRNA gene amplification (ΔCt), and the relative abundance of each gene of interest was finally calculated as 2^−ΔCt^.

### 2.4. Statistics

Only taxa forming at least 0.1% of total microbiota in either indoor or outdoor chickens were considered. Since we were always interested in the abundance of a particular microbiota member in indoor and outdoor chickens, the Mann–Whitney test was used to identify differently abundant taxa. Differences with *p* < 0.05 were considered as significant.

## 3. Results

### 3.1. Microbiota Composition of Hens from Indoor/Commercial and Outdoor/Backyard Poultry Flocks

**Figure 2:**
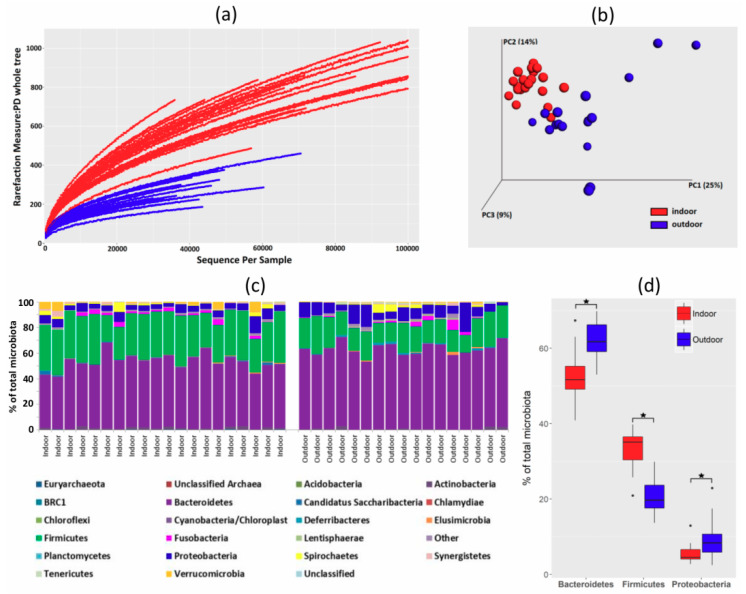
Microbiota of indoor and outdoor hens. (**a**) Rarefaction curves of individual hens from indoor, commercial flocks (red color) and outdoor, backyard flocks (blue color). (**b**) Weighted principal coordinate analysis (PCoA) analysis of caecal microbiota of individual hens from commercial flocks (red color) and backyard flocks (blue color). (**c**) Composition of caecal microbiota of individual hens from indoor and outdoor flocks at the phylum level. Of the majority phyla, microbiota of chickens from indoor commercial flocks was enriched for Gram-positive Firmicutes while microbiota of outdoor, backyard, chickens was enriched for Gram-negative Bacteroidetes and Proteobacteria (Panel (**d**)). * significantly different abundance in indoor and outdoor chickens by Mann–Whitney test, *p* < 0.05.

In total, 2,365,145 sequence reads were obtained for 37 analyzed samples with an average coverage of 63,922 reads per sample. Minimal and maximal sample read coverage were 30,021 and 153,409 reads, respectively (Table S2). Microbiota composition in backyard hens differed from microbiota of commercial hens. Rarefaction curves showed that microbiota of backyard hens consisted of a lower number of bacterial species (Figure 2A). Different microbiota composition was confirmed by weighted PCoA analysis, which separated commercial hens from backyard hens (Figure 2B). At the phylum level, microbiota of both indoor and outdoor hens was dominated by Bacteriodetes, Firmicutes, and Proteobacteria (Figure 2C). However, microbiota of outdoor hens was enriched for Bacteroidetes (62.41 ± 4.47 % of total microbiota in outdoor hens compared to 52.01 ± 6.27 % in indoor hens, *p* < 0.05) and Proteobacteria (9.33 ± 4.99% of total microbiota in outdoor hens compared to 5.47 ± 2.24% in indoor hens, *p* < 0.05). Higher abundance of Bacteroidetes and Proteobacteria occurred at the expense of Firmicutes, which were less abundant in outdoor hens (20.66 ± 4.41% of total microbiota in outdoor hens compared to 33.28 ± 5.11% in indoor hens, *p* < 0.05, Figure 2D).

Differences in microbiota of indoor and outdoor hens were further analyzed at the genus level. Out of 86 genera forming at least 0.1% of total microbiota, 43 genera were more abundant in the microbiota of indoor hens, and 10 genera were more abundant in the microbiota of outdoor hens. In agreement with Figure 2D, 27 genera enriched in indoor chickens belonged to phylum Firmicutes, while 6 genera dominating in outdoor chickens belonged to phyla Bacteroidetes or Proteobacteria (Table 1).

### 3.2. Real-Time PCR Quantification of Selected Genera

In selected genera, their differential abundance was verified by quantitative real-time PCR. *Anaerotruncus colihominis*, *Pseudoflavonifractor capillosus*, *Butyricicoccus pullicaecorum*, *Flavonifractor plautii*, and *Olsenella uli* were selected as species representing genera more abundant in microbiota of indoor chickens, and *Bacteroides mediterraneensis*, *Bacteroides coprophilus*, *Mucispirillum schaedlerii*, and *Marseilla massiliensis* (this species was selected as representative of Prevotellaceae) were selected as species representing genera more abundant in microbiota of outdoor chickens.

**Figure 3:**
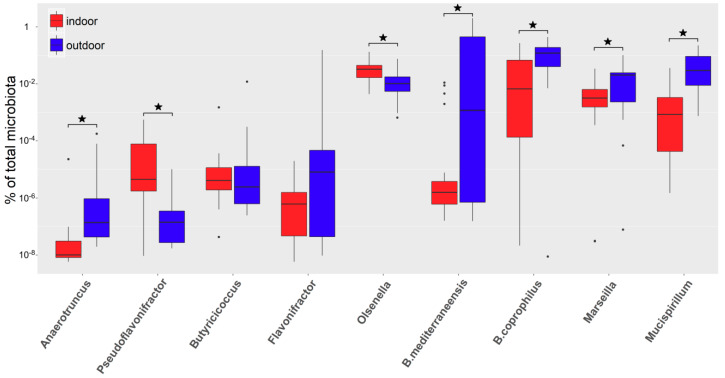
Real-time PCR quantification of selected species representing genera identified as differentially abundant in the microbiota of indoor (red boxes) and outdoor (blue boxes) chickens by 16S rRNA gene sequencing. * significantly different by Mann–Whitney test, *p* < 0.05.

Real-time PCR confirmed the results of 16S rRNA sequencing for *Pseudoflavonifractor capillosus* and *Olsenella uli* as being more abundant in the microbiota of indoor chickens and for *Bacteroides mediterraneensis*, *Bacteroides coprophilus*, *Mucispirillum schaedlerii*, and *Marseilla massiliensis* as being more abundant in the microbiota of outdoor chickens. Real-time PCR did not confirm significant differences in abundance for *Butyricicoccus pullicaecorum* and *Flavonifractor plautii*, and conflicting results were recorded for *Anaerotruncus colihominis*. Although 16S rRNA sequencing predicted the genus *Anaerotruncus* as more abundant in indoor chickens, *Anaerotruncus colihominis* was significantly more represented in the microbiota of outdoor chickens (Figure 3).

### 3.3. Real-Time PCR Quantification of Antibiotic Resistance Genes

**Figure 4:**
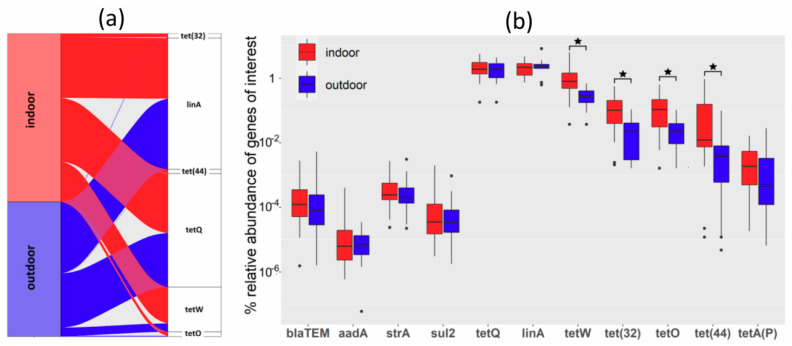
Abundance of antibiotic resistance genes in microbiota of commercial and backyard hens. (**a**) Abundance of the most common antibiotic resistance genes in total microbiota and their association with indoor or outdoor chickens. (**b**) Abundance of all analyzed antibiotic resistance genes in total microbiota in hens from indoor, commercial poultry flocks (red color) and outdoor, backyard poultry flocks (blue color). * significantly different by Mann–Whitney test, *p* < 0.05.

Use of antibiotics for therapeutic purposes is relatively common in chickens in commercial production. On the other hand, owners of backyard outdoor chickens use antibiotics very rarely. This is why we tested the abundance of antibiotic resistance genes in microbiota of indoor and outdoor chickens. Using quantitative real-time PCR, the abundance of *bla_TEM_*, *aadA*, *strA*, *sul2*, *tetA(P)*, *tetO*, *tetQ*, *tet(32)*, *tet(44)*, *tetW*, and *linA* was determined. Of all tested antibiotic resistance genes, *tetW*, *tetO*, *tet(32)*, and *tet(44)* were significantly more frequent in the microbiota of hens from commercial flocks than in the microbiota of outdoor, backyard hens. The difference in abundance of antibiotic resistance genes was therefore detected in genes commonly found in Firmicutes (*tetO*, *tet(32*)*, tet(44)*, *tetW*) while the abundance of antibiotic resistance genes characteristic for Bacteroidetes (*tetQ* and *linA*) or Proteobacteria (*bla_TEM_*, *aadA*, *strA*, *sul2*) was similar in both commercial and backyard hens (Figure 4).

## 4. Discussion

In this study, we compared the composition of caecal microbiota in egg-laying hens kept indoors in commercial farms and hens kept outdoors in the backyards of private owners (Figure 1). Similar to previous reports, gut microbiota in both groups of chickens was dominated by representatives from phyla Bacteroidetes, Firmicutes and Proteobacteria [10,11,12,13]. The fact that we did not confirm results from 16S rRNA sequencing by real-time PCR of selected species belonging to given genera was likely caused by the fact that different species and clones within particular species are summed up at the genus level. Further comparison with previous reports is complicated since these were performed with indoor and outdoor broilers, i.e., chickens before reaching sexual maturity [12,14,15], or the microbiota of outdoor hens was compared with microbiota of commercial broilers [16]. This is probably the reason why Ocejo et al. reported a higher microbial diversity in free-range broilers compared to indoor broilers [12], while we recorded lower species diversity in the microbiota of outdoor hens. Young chickens are highly susceptible to colonization with different microbiota members [17,18,19], and exposure of free-range chickens to greater microbiota sources present in an outdoor environment could result in microbiota with transiently higher diversity. On the other hand, our analysis was performed in adult hens with already established microbiota shaped by external conditions including diet what for example excludes also potential effect of human handlers on hen’s gut microbiota. Feed in commercial production is designed to meet all the chicken’s dietary requirements and is rich in available energy, balanced in carbohydrates, protein, fat content, and supplements like vitamins, minerals, and other growth factors. The same optimal balance of nutrients in the feed of outdoor chickens is unlikely. Since the feed is also a growth substrate for gut microbiota, the broader the nutrient sources and a higher the substrate availability may result in a higher diversity of microbiota in indoor hens.

Although Ocejo et al. reported a higher representation of *Bacteroides* in indoor chickens than in free-range [12], two other studies reported the same result as we recorded, i.e., that Bacteroidetes were more abundant in the microbiota of outdoor rather than in indoor chickens [14,16]. A likely explanation might be the potential of Bacteroidaceae and Prevotellaceae to digest complex polysaccharides of plant origin [20]. Microbiota enrichment for Prevotellaceae has been reported even in humans from rural areas whose diet is rich in polysaccharide fiber of plant origin [21,22]. Microbiota of outdoor chickens was enriched also for Proteobacteria, specifically genera *Succinatomonas* or *Sutterella*. The function of these bacteria in gut microbiota is not well understood but these genera were reported as common in microbiota of outdoor chickens also in previous studies [12,16]. The increased abundance of Bacteroidetes and Proteobacteria occurred at the expense of Firmicutes. We have shown recently that the genome of isolates from phylum Firmicutes is rich in genes encoding enzymes for oligosaccharide metabolism but genes for the metabolism of complex polysaccharides are less frequent in the genomes of Firmicutes compared to Bacteroidetes [20]. The lower genetic potential of chicken Firmicutes to digest complex polysaccharides of plant fibre origin may therefore explain their lower abundance in outdoor hens and easily digestible mono- and oligo-saccharides present in commercial feed may further contribute to Firmicutes enrichment in the microbiota of commercial hens.

Antibiotics are quite commonly used in commercial production while owners of backyard chickens rarely use antibiotics. This explains the lower abundance of antibiotic resistance genes in the microbiota of outdoor chickens. Similar data were reported also by Ferairo et al. [16]. Due to the low abundance of Enterobacteriaceae in the microbiota of adult hens, the frequency of genes common to this family was quite low. Interestingly, *tetQ* and *linA* genes characteristic of Bacteroidetes [23] were the most frequent and equally abundant antibiotic resistance genes in microbiota of indoor and outdoor chickens. On the other hand, the abundance of *tetO*, *tet(32)*, *tet(44)*, and *tetW* genes characteristic of Firmicutes [24,25,26] was lower but with significant differences in microbiota of indoor and outdoor chickens. Horizontal gene transfer in Bacteroidetes is commonly associated with conjugative plasmids and transposons [23,27,28], and since conjugative transfer is highly effective, even a single positive isolate may act as a highly efficient donor of antibiotic resistance for the remaining members of the population. On the other hand, Firmicutes pick up foreign DNA by inducing competence by transformation [29,30]. However, since transformation is less efficient than conjugation, the spread of antibiotic resistance genes from a single positive isolate is not so effective, and these genes are less common throughout the population.

## 5. Conclusions

The initial aim of this study was to identify microbiota members enriched in the microbiota of backyard hens since these may represent novel probiotics, which are for different reasons unavailable for commercially raised hens. However, when thinking critically, even the genera more abundant in the microbiota of commercial hens can be considered as having probiotic potential since their higher abundance in commercial hens indicates their higher adaptation for indoor hens’ genetics, feed, and living environment. Despite this, rather surprisingly, we realized that microbiota members that (i) were present at high but similar abundance in both indoor and outdoor hens but (ii) are usually absent from the microbiota of commercially hatched chickens during the first days of their life [1] and additionally (iii) efficiently colonize the caecum of newly hatched chicks if presented experimentally [31] are those that should be initially considered as probiotics, since such genera and species belong to the chicken intestine irrespective of chicken genetics, feed composition or living environment.

## Figures and Tables

**Table 1:** Bacterial genera significantly more abundant either in microbiota of indoor or outdoor chickens.

Phylum	Genus	In/Out ^#^	Indoor *	Outdoor *	*p* Value
Actinobacteria	**Olsenella** ^$^	2.59	0.71	0.27	*p* < 0.01
Bacteroidetes	Tannerella	2.94	0.40	0.14	*p* < 0.01
Bacteroidetes	Flavobacteriaceae;Other	2.55	0.45	0.18	*p* < 0.01
Bacteroidetes	Phocaeicola	2.49	0.17	0.07	*p* < 0.01
Bacteroidetes	Odoribacter	1.85	0.49	0.27	*p* < 0.01
Bacteroidetes	Bacteroidetes;Other	1.66	8.53	5.13	*p* < 0.01
Bacteroidetes	Porphyromonadaceae;Other	1.50	3.63	2.42	*p* < 0.01
Bacteroidetes	Bacteroidales;Other	1.46	7.35	05.3	*p* < 0.05
Candidatus Saccharibacteria	Saccharibacteria_genera_incertae_sedis	5.15	0.51	0.10	*p* < 0.01
Euryarchaeota	Methanobrevibacter	4.92	0.19	0.04	*p* < 0.01
Firmicutes	Acetanaerobacterium	6.29	0.19	0.03	*p* < 0.01
Firmicutes	Ethanoligenens	5.64	0.10	0.02	*p* < 0.01
Firmicutes	**Anaerotruncus**	04.12	0.25	0.06	*p* < 0.01
Firmicutes	Peptococcus	3.60	0.13	0.04	*p* < 0.01
Firmicutes	Firmicutes;Other	3.35	0.37	0.11	*p* < 0.01
Firmicutes	Acidaminococcaceae;Other	3.28	0.12	0.04	*p* < 0.01
Firmicutes	Coprococcus	03.7	0.14	0.04	*p* < 0.01
Firmicutes	Eubacterium	3.00	0.12	0.04	*p* < 0.01
Firmicutes	**Flavonifractor**	2.94	1.17	0.40	*p* < 0.01
Firmicutes	Ruminococcus2	2.81	2.67	0.95	*p* < 0.01
Firmicutes	**Pseudoflavonifractor**	2.77	0.95	0.34	*p* < 0.01
Firmicutes	Clostridium XI	2.63	0.32	0.12	*p* < 0.01
Firmicutes	Clostridium IV	2.56	0.72	0.28	*p* < 0.01
Firmicutes	Ruminococcus	2.54	0.15	0.06	*p* < 0.01
Firmicutes	Lachnospiraceae;Other	2.47	2.98	1.20	*p* < 0.01
Firmicutes	Ruminococcaceae;Other	2.41	4.49	1.86	*p* < 0.01
Firmicutes	**Butyricicoccus**	2.38	0.41	0.17	*p* < 0.01
Firmicutes	Roseburia	2.27	0.16	0.07	*p* < 0.01
Firmicutes	Erysipelotrichaceae_incertae_sedis	02.5	0.14	0.07	*p* < 0.05
Firmicutes	Subdoligranulum	1.90	0.46	0.24	*p* < 0.01
Firmicutes	Oscillibacter	1.89	1.25	0.66	*p* < 0.01
Firmicutes	Clostridiales;Other	1.78	3.39	1.90	*p* < 0.05
Firmicutes	Blautia	1.77	0.27	0.15	*p* < 0.05
Firmicutes	Lachnospiracea_incertae_sedis	1.54	0.13	0.08	*p* < 0.05
Firmicutes	Clostridium XlVa	1.43	0.92	0.64	*p* < 0.05
Firmicutes	Clostridium XlVb	1.38	0.36	0.26	*p* < 0.05
Firmicutes	Clostridia;Other	1.26	0.12	0.09	*p* < 0.05
Proteobacteria	Vampirovibrio	3.99	0.49	0.12	*p* < 0.05
Proteobacteria	Parasutterella	1.46	0.43	0.29	*p* < 0.05
Spirochaetes	Spirochaetaceae;Other	1.78	0.27	0.15	*p* < 0.05
Verrucomicrobia	Opitutae;Other	199.31	0.38	0.00	*p* < 0.01
Verrucomicrobia	Subdivision5_genera_incertae_sedis	9.65	01.1	0.10	*p* < 0.01
Verrucomicrobia	Akkermansia	9.50	0.11	0.01	*p* < 0.01
Bacteroidetes	**Bacteroides**	0.59	15.57	26.51	*p* < 0.01
Bacteroidetes	**Prevotella**	0.25	02.4	08.9	*p* < 0.05
Deferribacteres	**Mucispirillum**	0.29	0.20	0.69	*p* < 0.01
Firmicutes	Dialister	0.04	0.04	01.5	*p* < 0.01
Lentisphaerae	Victivallis	0.30	0.16	0.54	*p* < 0.05
Proteobacteria	Succinatimonas	0.44	0.12	0.27	*p* < 0.01
Proteobacteria	Sutterellaceae;Other	0.38	0.08	0.22	*p* < 0.01
Proteobacteria	Sutterella	0.21	0.26	1.21	*p* < 0.01
Proteobacteria	Anaerobiospirillum	0.13	0.29	2.32	*p* < 0.01
Tenericutes	Asteroleplasma	0.26	0.15	0.59	*p* < 0.05

* percentage average abundance in total microbiota of indoor or outdoor chickens. # ratio of abundance of a given genus in indoor and outdoor hen microbiota. $ differentially abundant genera in bold were selected for verification by real-time PCR specific for a particular species within the given genus. If underlined, real-time PCR data confirmed 16S rRNA sequencing results.

## Supplementary Materials

The following are available online at https://www.mdpi.com/2076-2607/8/5/767/s1, Table S1. List of primers used in this study; Table S2. OTU (Operational Taxonomic Unit) table limited to OTUs forming at least 0.1 % of total microbiota in at least one of tested hens.
